# Recommendations to Improve Healthcare Service Provision for Cognitive Impairment in People With Parkinson's Disease: A Mixed Methods Study of the Lived Experience Expert Perspective

**DOI:** 10.1111/hex.70530

**Published:** 2026-03-15

**Authors:** Deepa Sriram, Dana Pourzinal, Daniel X. Bailey, Deborah Brooks, Kirstine Shrubsole, Jihyun Yang, Neil Page, Emily McCann, Elton H. Lobo, James M. King, Leander K. Mitchell, Nancy A. Pachana, Nadeeka N. Dissanayaka

**Affiliations:** ^1^ UQ Centre for Clinical Research The University of Queensland Herston Queensland Australia; ^2^ Centre for Health Services Research The University of Queensland Herston Queensland Australia; ^3^ Australian Centre for Health Services Innovation (AusHSI) and Centre for Healthcare Transformation, School of Public Health and Social Work Queensland University of Technology (QUT) Brisbane Queensland Australia; ^4^ Queensland Aphasia Research Centre The University of Queensland Brisbane Queensland Australia; ^5^ School of Psychology The University of Queensland Brisbane Queensland Australia

## Abstract

**Background:**

Cognitive impairment, including dementia, is one of the most important nonmotor symptoms of Parkinson's disease (PD). It lowers quality of life and impacts health and economic outcomes for individuals living with PD, their families, and society. Poor recognition and management of cognitive impairment and dementia in PD highlights the need for improved diagnostic and postdiagnostic care pathways. We aimed to inquire about current health services for cognitive evaluation in Australia from people with lived experience of PD. The objective was to derive recommendations for best practice guidelines.

**Methods:**

This two‐stage exploratory sequential mixed‐method study utilised qualitative and quantitative methods. Stage 1 conducted seven online focus groups exploring the experiences of neuropsychological assessment, diagnosis, and post‐diagnostic support for cognitive impairment and dementia in PD. Transcripts were analysed using deductive and inductive thematic analysis and recommendations were derived from this data. Stage 2 involved a national survey of these recommendations to ascertain agreement using a 5‐point Likert scale. Recommendations meeting ≥ 70% agreement, median rating ≥ 4, and inter quartile rating (IQR) ≤ 1 were deemed acceptable for inclusion in the guidelines.

**Results:**

Focus groups included people with PD (PwPD) with subjective cognitive decline (PD‐SCD, *n* = 6), mild cognitive impairment (PD‐MCI, *n* = 3), dementia (PDD, *n* = 3), and carers (*n* = 3). Findings resulted in the formulation of 25 recommendations from four overarching categories and with several inter‐related themes: (1) Pre‐assessment (clinicians' reluctance to assess; referrals; informed choice), (2) assessment (delivery of assessment; telehealth), (3) diagnosis (need for transparency; focused discussion; time to discuss), and (4) postdiagnostic care (follow‐up assessment; information in plain language; advocating for PwPD). The national survey (*n* = 69 PwPD, *n* = 12 carers) found that all recommendations except one demonstrated high agreement (≥ 88%, median rating ≥ 4, IQR ≤ 1). Delivery of a cognitive diagnosis on the same day as cognitive testing was the only area that did not achieve consensus.

**Conclusion:**

We identified critical gaps in the diagnosis and management of cognitive symptoms within clinical services, and the subsequent implications for PwPD and their carers. These results provide a lived experience perspective to the development of best practice guidelines for cognitive evaluation in PD.

**Public Contribution:**

The project was endorsed by our Consumer and Community Involvement Group (CCIG), a research advisory board consisting of people with lived experience of Parkinson's Disease and dementia, including those caring for PwPD. The CCIG identified initial need for the research project, were involved in refining the focus group topic guide and refining the recommendations for the national survey.

## 1 Introduction

Cognitive impairment is an impactful nonmotor manifestation in Parkinson's disease (PD) [1]. Cognitive impairment in PD ranges in severity from subjective cognitive decline (PD‐SCD) to mild cognitive impairment (PD‐MCI) and dementia (PDD) [2]. PD‐SCD refers to a self‐reported decline in cognitive abilities despite no indication of performance deficits on standardised cognitive tests [3]. PD‐MCI is characterised by objective cognitive impairment with functional independence [4], whereas PDD is defined by objective cognitive impairment and the loss of functional independence or impairment in activities of daily living [5]. Dementia develops in over 80% of people living with PD (PwPD) within 20 years of a PD diagnosis [6]. Dementia in PD is associated with an increased risk of falls, reduced quality of life, greater burden on carers, earlier institutionalisation, and increased financial strain [7]. Despite this, current guidelines for the diagnosis and evaluation of cognitive disorders in PD are outdated and do not include input from people with lived experience of the condition.

Early identification of cognitive disorders is critical to prevent missed opportunities for early intervention, effective treatment, future planning and support [1]. In Australia, PD is typically diagnosed in general neurology services, specialised movement disorders clinical services or in geriatric services. Clinical attention is primarily directed towards managing motor symptoms, and opportunities and resources for cognitive evaluation are often scarce. This challenge is present globally, where the management of cognitive disorders in PD is inconsistent across clinics, leaving many PwPD to navigate gaps in the healthcare system without adequate support [8, 9]. Yet, PwPD report cognitive decline to be one of their most significant concerns [1, 9], highlighting a critical need for more structured and comprehensive cognitive evaluation and management practices in PD care [1, 10].

People with cognitive disorders in PD often report ‘falling through the gaps’ in the healthcare system [9], a problem supported by the fact that existing guidelines for diagnosis, evaluation, and management lack specific input from individuals with lived experience [4, 5, 11]. The World Health Organization (WHO) emphasises the importance of meaningful engagement with people with lived experience to enhance relevant policies, programs, and services [12]. Therefore, the present study sought to identify recommendations for the diagnosis, evaluation, and management of cognitive disorders in PD that were developed and endorsed by PwPD. Findings from the present study will inform a larger program of research to develop best practice guidelines for cognitive disorders in PD.

## 2 Methods

**Figure 1:**
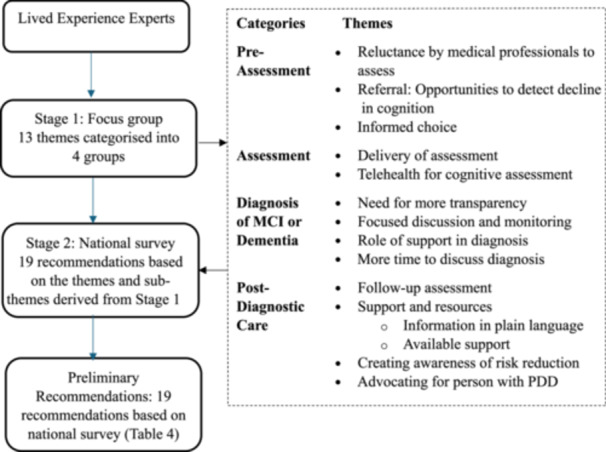
Flowchart of the two‐staged study.

This study was underpinned by a participatory approach, ensuring that people with lived experience were meaningfully involved throughout the research process. It followed a two‐stage exploratory sequential mixed‐methods approach (Figure 1) to develop lived experience expert recommendations following a published methodology [13]. Stage 1 was an exploratory qualitative study, utilising focus group discussions with PwPD and their carers to understand their experiences accessing healthcare services for cognitive complaints. Specifically, participants were asked to discuss their experiences receiving neuropsychological assessment, diagnoses, and post‐diagnostic care for cognitive disorders. Participants also discussed potential strategies to improve their overall clinical experience. These findings were used to develop potential recommendations. An exploratory qualitative methodology was used in Stage 1 due to the paucity of research in this area, collected from people with varying levels of impairment. The Consolidated Criteria for Reporting Qualitative Research (COREQ) checklist [14] was used for reporting.

Stage 2 was a confirmatory Australia‐wide survey of PwPD and their carers that evaluated their endorsement of the potential recommendations for inclusion in guidelines for cognitive disorders in PD. The survey methodology in Stage 2 served to validate the feedback obtained from a small sample within a broader population of PwPD who had undergone a neuropsychological assessment, regardless of the outcome.

### 2.1 Consumer and Community Involvement Group (CCIG)

The CCIG comprised 13 people with lived experience of neurodegenerative diseases across Australia. This includes PwPD, Alzheimer's disease, frontotemporal dementia, dementia with Lewy bodies, their carers, and members of community organisations, for example, Lions club. Members varied in age, experience, and geographical state, and the insights provided were invaluable. The CCIG played a critical advisory role throughout the study. The CCIG contributed to the development of study materials, recruitment processes, and the review and refinement of recommendations based on their lived experience. Monthly meetings were held to discuss all stages of the study, from conception to preliminary results, with study documents also circulated via email for review and feedback. This ensured that the study was informed by community and consumer perspective at all stages of the study.

### 2.2 Sampling and Eligibility

Lived experience experts were categorised into three participant groups included people living with PD‐SCD, PD‐MCI, PDD, respectively, based on self‐reported diagnoses from prior neuropsychological evaluations related to PD. Carers included in this study were informal carers of PwPD. Any person with a diagnosis of PD or carer of someone living with PD able to communicate in English was eligible to participate in both stages. Importantly, only those with experience of neuropsychological evaluation related to PD were eligible to take part in the focus group discussions. Participants were recruited using purposive sampling via the CCIG, PD community groups and PD and carer‐related organisations (e.g., Parkinson's Australia). Ninety‐nine potential participants completed an online expression of interest for the group discussion and/or national survey. We also utilised ‘snowballing’ techniques by encouraging participants to recommend the study to eligible contacts, both for the focus groups and the national survey. Focus group and survey sample sizes were guided by the Australia Dementia network (ADNET) methodology for developing best practice guidelines [13]. We aimed to conduct at least two focus groups per participant group to ensure a diversity of perspectives and sufficient coverage of experiences related to cognitive assessment and management in PD. For the survey, a sample size of approximately 100 was considered ideal to obtain broad input from PwPD and carers, consistent with the ADNET approach to guideline development [13].

### 2.3 Recruitment Procedures

Potential participants who had expressed interest to participate were contacted by email and/or telephone to confirm their interest, provide study information, and screen for eligibility (e.g., diagnosis of Parkinson's disease and cognitive status). A separate verbal evaluation was conducted for each participant with PDD and PD‐MCI to confirm their ability to provide informed consent. The Evaluation to Sign Consent is a five‐item measure which can determine if a person living with cognitive impairment or dementia, has the capacity to understand the process, requirements and risks of research to provide their own informed consent; all these participants were able to provide their own informed consent [15]. Participants living with PD‐MCI and PDD were given the option to attend the focus group with a support person (carer/family member).

For the online survey, a survey link with an embedded consent form was distributed to: (i) PwPD and carers who expressed interest to participate, (ii) nationwide PD‐related organisations, (iii) carer‐led organisations, and (iv) participants from previous PD‐related research who expressed interest in further research participation. Participants had the option to receive assistance from their care partner or support person to complete the survey. Informed consent was obtained from all focus group and survey participants.

### 2.4 Data Collection and Analysis

#### 2.4.1 Stage 1 (Focus Groups)

Topic guide (Table 1) development was informed by research aims and existing literature [16], which was reviewed and refined by the CCIG and multidisciplinary study team to ensure critical issues were addressed and appropriate language was used. The multidisciplinary study team included members with expertise in geriatric psychology, neurology, and allied health. Focus groups were conducted separately for each participant group (PD‐SCD, PD‐MCI and PDD) and were restricted to a maximum of four participants per group to ensure active participation. Data were collected from May to August 2024 via online platforms (Zoom Video Communications or Microsoft Teams). Focus groups were facilitated by three postdoctoral researchers with experience in qualitative studies and PD cognition (D.X.B., K.S. and J.Y.) using a semistructured guide which was adapted to support participants with cognitive impairment. Specialised communication training was provided to interviewers by a speech pathologist experienced in working with PwPD and conducting qualitative research. Duration of the focus groups ranged from 45 to 60 min. Facilitator names and discussion questions were displayed on‐screen for clarity. Participants could pause or reschedule the session if fatigued or distressed. Two facilitators attended each session: one led the focus group questions and guided the discussion, while the other documented attendance and field notes, provided communication or cognitive support as needed, and was available to speak with participants in a break‐out room should any distress arise (although no such instances occurred). Focus groups were recorded and transcribed verbatim by a professional service under a confidentiality agreement, using only de‐identified audio recordings. Data familiarisation and preliminary analysis took place alongside data collection for ongoing assessment of information collected. Data collection ceased once the researchers determined that data were sufficient to effectively address the study objectives [17].

**Table 1:** Focus group guide.

Focus group with lived experience experts
Basic demographic information:
− Gender, age
Parkinson's disease relevant information:
− Year diagnosis with Parkinson's disease, any diagnosis of cognitive decline/Parkinson's dementia
Experience of attending clinics/assessment:
− Details of which clinicians they see and at what intervals − Who referred them to have a neuropsychological evaluations and why
Experience of receiving a diagnosis (if any):
− Who delivered the diagnosis and how was this delivered? − What information related to the diagnosis did you receive?
Experience of postevaluations and postdiagnostic support:
− Were you referred on to any clinicians after diagnosis? Who made this referral? − Have you had further cognitive evaluations since your diagnosis? At what time interval?
Recommendations for positive or negative experiences:
− Based on their experience of having undergone the assessment (and diagnosis) process are there any parts of the process that should be changed? − Are there any experiences or actions that you would recommend people who will undergo or are undergoing the assessment process should do?

A combination of deductive and inductive approaches using an interpretive framework were deemed the most suitable for understanding of cognitive evaluation in PD in line with the research aims [17, 18]. An interpretive framework helped make sense of participant experiences in the context of how assessments are understood, used, and experienced in real‐world settings [19]. Deductive thematic analysis was utilised where the research aims and questions guided the identification of key categories. Inductive coding allowed new themes to emerge organically from the data [20]. Thematic analysis is a form of pattern recognition within the data, highlighting similarities and differences, and generating deeper insights of the phenomenon: cognitive evaluation in PD [20, 21]. NVivo Release 14.23.4 was used for coding, which was conducted independently by two investigators (D.S. and D.X.B.). The investigators (D.S. and D.X.B.) met to review, define, and name the themes. Meetings were also conducted with a third investigator (D.P.) with expertise in PD cognition to further refine the themes. Finally, themes were presented to the multidisciplinary study team and CCIG members for peer debriefing and to confirm interpretation of the findings.

#### 2.4.2 Stage 2 (National Survey)

Recommendations for diagnostic and postdiagnostic processes for cognitive disorders in PD were developed based on themes derived from the focus group analysis. Recommendations were then reviewed and refined by our multidisciplinary study team and the CCIG, who also contributed suggestions based on their lived experience. Final recommendations were compiled in an online survey and distributed through relevant recruitment avenues from October to December 2024 via a secure web‐based software platform, REDCap (Research Electronic Data Capture). The online survey included instructions and definitions of key clinical terms (e.g., cognition, cognitive impairment and neuropsychological assessment). Participants were asked to rate each recommendation on a five‐point Likert scale (1 = strongly disagree to 5 = strongly agree). Participants had 2 weeks to complete the survey, with a reminder sent after 1 week and again nearer the deadline.

Data were analysed using IBM SPSS (version 30.0.0). Descriptive statistics, including frequencies, percentages, median and inter‐quartile range (IQR) were computed for each item. In line with the ADNeT methodology, any recommendation that achieved ≥ 70% agreement was considered to be endorsed by lived experience experts [13]. To ensure additional rigour, recommendations were also required to have a median score of ≥ 4/5 and an IQR ≤ 1.

## 3 Results

### 3.1 Stage 1: Focus Groups

#### 3.1.1 Sample Characteristics

In total, seven focus groups were conducted (*N* = 15):

Two with people living with PDD.

Focus Group 1: two people with PDD, and one carer.
Focus Group 2: one person with PDD, and one carer.

Two with people living with PD‐MCI.

Focus Group 3: one person with PD‐MCI, and one carer.
Focus Group 4: two people with PD‐MCI.

Three with people living with PD‐SCD (each comprising two participants with SCD).

Table 2 describes the participant characteristics.

**Table 2:** Focus group participant characteristics (*N* = 15).

Participant group	No. (%)	Participant characteristics				Parkinson's diagnosis duration^a^	Cognitive impairment duration^a^
		Gender	*n*	Age range	*n*	Range in years	Range in years
Parkinson's disease with dementia (PDD)	3 (20)	Male	3	55–60 70–75	2 1	3–6	3–5
Parkinson's disease with mild cognitive impairment (PD‐MCI)	3 (20)	Male	3	50–55 60–65	1 2	4–13	0.25–8
Parkinson's disease with subjective cognitive decline (PD‐SCD)	6 (40)	Male Female	3 3	50–55 60–65 65–70	2 1 3	3–9	N/A
Carer	3 (20)	Male (son) Female (partner)	1 2	25–30 55–60	1 2	N/A	N/A
State							
Queensland	7 (47)						
New South Wales	5 (33)						
Victoria	1 (7)						
Australian Capital Territory	1 (7)						
Western Australia	1 (7)						

a Duration of diagnosis as of 2025.

#### 3.1.2 Results

The initial coding categories (based on the discussion guide) formed the basis on which the themes were organised. Figure 1 represents the different stages of the study, as well as the summarised categories and themes from Stage 1.

Each theme is described below with representative participant quotes.

##### 3.1.2.1 Category 1. Preassessment

###### 3.1.2.1.1 Reluctance to Assess

Focus group participants reported that the cognitive features of PD were often not acknowledged by clinicians at the time of PD diagnosis. Some participants and/or their carers took it upon themselves to ask for neuropsychological evaluation as there was reluctance by the medical professionals to assess for cognition in PwPD.

Because the neurologist was reluctant to entertain that there could be cognitive decline or dementia, we actually ended up going to a geriatrician that was instigated by myself.

PDD_Carer_P4 (PDD: M, 55; Carer of the person with PDD: F, 55)

Participants suggested that providing PwPD information on what to expect across the cognitive impairment and dementia continuum would assist with forward planning and supported baseline cognitive screening at the time of PD diagnosis.

It would be good to be able to start talking about possible other complaints and how they can start and how you can identify them. So do some forward planning.

PD‐SCD_P2 (M, 50)

I would prefer to have it—have something [cognitive screening], I'm aware of the importance of the movement side of it. But the cognition side of it, being a person who lives on my own is probably more a—a bigger concern for me to going forward as my Parkinson's progresses.

PD‐SCD_P7 (F, 68)

###### 3.1.2.1.2 Referral

Any noticeable change in cognition was seen as an opportunity for referral for neuropsychological evaluation. Participants agreed that allied health professionals, with whom they had more regular contact, had greater opportunities to detect cognitive impairment, and dementia, and therefore should be able to refer for neuropsychological evaluation.

Whereas the allied health professionals when you're in regular contact with them, they have a better baseline of assessment for changes. Allied health professionals that's already seeing them, the baseline for symptomatic tracking is more accurate.

PDD_P3 (M, 58)

###### 3.1.2.1.3 Informed Choice

In‐line with the information for forward planning at the time of PD diagnosis, participants discussed the need for clear information about cognitive impairment, and dementia, neuropsychological evaluations, and the implications and benefits of the evaluations. Some participants expressed that they wanted to feel in control and be able to make informed decisions about their cognitive evaluation.

Having it contextualised so that you know why you're doing it [neuropsychological assessment], what you're doing it for, what the outcome is, what the implications are, and the benefits as a result. And then the strategies, associated with correction or dealing with.

Carer‐PD‐MCI_P11 (Carer: M, 28; PD‐MCI: M, 60)

Awareness of the health professionals involved in PD care was considered important. Some participants expressed a preference for a multidisciplinary team throughout their PD journey. A clear understanding of the neuropsychological evaluation and care process was deemed essential.

What I would certainly favour, is a multidisciplinary approach, where you could be going to a hospital and be assessed and you talk to a neurologist, psychologist, an OT, and you come up with a treatment plan.

PD‐SCD_P9 (M, 67)

##### 3.1.2.2 Category 2. Neuropsychological Assessment

###### 3.1.2.2.1 Delivery of Assessment

Participants emphasised the critical importance of the timing of evaluations, delivery of diagnoses, and the familiarity of the clinician. Assessments were complicated by fluctuations in response to medication. While participants perceived the evaluations as rigid and confrontational, they suggested that having a familiar clinician conduct it could potentially alleviate the anxiety they experienced.

I'm sitting in a hospital, in a sterile room and I'm feeling anxious, and you want me to remember stuff. So that didn't really work for me. It [assessment] would be much better if it was someone that was more familiar, for sure.

PD‐MCI_P5 (M, 64)

Postponing the diagnosis following an extensive neuropsychological assessment also enables a more careful interpretation of results, minimises immediate emotional distress, and enhances the accuracy and sensitivity of clinical conclusions.

###### 3.1.2.2.2 Telehealth for Cognitive Assessment

The participants who lived in regional areas discussed the difficulties in accessing clinicians, especially specialists. Some of the PwPD reported having utilised telehealth services. Telehealth was found to be beneficial for those in regional areas though support may be required for PwPD without strong computer skills or with cognitive impairment.

Telehealth is certainly embedded in the medical, practitioners’ area, I don't see why not for cognitive assessment.

PD‐SCD_P2 (M, 50)

##### 3.1.2.3 Category 3. Diagnosis

###### 3.1.2.3.1 Transparency

Participants indicated that they would prefer to know if they met the criteria for dementia. Having an explicit diagnosis was preferred rather than ‘some cognitive decline’, and not being given a firm diagnosis was a concern raised by participants. They perceived that having a clear diagnosis explained their experiences (e.g., forgetfulness), and was often accompanied with a sense of relief, despite being a negative diagnosis. Similarly, participants highlighted the need for transparency during the diagnosis consultation and including carers in diagnostic discussions.

Think if it was a blood test, you would see it. Why are the neuropsych assessments so different when it's a life changing diagnosis, not only for the person but also for their family? and we are locked out.

Carer‐PDD_P4 (PDD: M, 55; Carer: F, 55)

###### 3.1.2.3.2 Focused Discussion and Monitoring

A more focused in‐depth discussion about what a cognitive impairment or dementia diagnosis would mean for different areas of their life was deemed important by some PwPD.

I guess for me, probably if [consultation about cognition] was specific and there was a bit more focused mention to [cognition] and monitoring of it, a more detailed manner, I think it would be helpful.

PD‐MCI_P6 (M, 52)

Some participants, however, believed that the choice for detailed discussion should be with the PwPD rather than the clinician.

I had a long appointment but, because there is so much information given to you at that first appointment, a lot of that probably just goes straight over your head. You don't absorb it, because there is so much information.

PD‐MCI_P5 (M, 64)

###### 3.1.2.3.3 Role of Support in Diagnosis

As aforementioned, the majority of participants agreed that a diagnosis should be delivered with a support person present. They emphasised that having this support was essential and also suggested referrals to formal services like social workers, counsellors, and allied health professionals to improve current practice.

Because especially if you're getting a diagnosis of cognitive impairment, and you get some advice afterwards, how much of it are you going to remember, I think if it was recommended that you take someone with you, that would be a good thing…let's put you in touch with a social worker who can run you through with. These are things that you might want to think about. Perhaps you want some counselling. Perhaps you need some support… This needs to be done.

Carer‐PD‐MCI_P11 (Carer: M, 28; PD‐MCI: M, 60)

###### 3.1.2.3.4 More Time to Discuss

In line with the focused discussion, for the PwPD, having a clinician who could spend time to clearly and respectfully explain the diagnosis of PD‐MCI or PDD, was very important.

His bedside manner was shocking. Yeah, someone who had more time to discuss with me, what it meant and then what I could do about it, would be good.

SCD_P1 (M, 63)

Participants shared the opinion that having awareness of a clear pathway, and what the short, medium, and long‐term outlook would be like, was key to their PD journey.

##### 3.1.2.4 Category 4. Post Diagnostic Care

###### 3.1.2.4.1 Follow‐Up Assessment

A desire for more frequent testing, with greater emphasis on cognition in their care plans, was expressed by PwPD and their carers. Having focused testing would enable monitoring progress of the specific areas of concern.

I think that they [neuropsychological assessment] need to be more frequent, but sort of focusing in on particular, weakness area.

Carer‐PD‐MCI_P11 (Carer: M, 28; PD‐MCI: M, 60)

###### 3.1.2.4.2 Support and Resources

####### 3.1.2.4.2.1 Information in Plain Language

Being given information on available resources and support groups was deemed crucial for postdiagnostic care of cognitive disorders in PwPD. Receiving information related to cognitive evaluation, diagnosis, or care written in plain English and using simple terminology was considered very important by both PwPD and their carers.

We go to the doctor and come out and he'd say what? What did he just say? What was that all about? And it wasn't necessarily the cognitive decline. It was the terminology that was being used, that he was not familiar with.

Carer‐PDD_P14 (Carer: F, 56; PDD: M, 71)

####### 3.1.2.4.2.2 Available Support

Participants found support groups valuable for understanding shared hardships and exchanging knowledge, treatment, and coping strategies. They also noted the importance of being informed about where to access resources and support.

so, it was a turning point, information related to the diagnosis. Not a lot of information and resources were given. I've googled it since, guess for me and my nature I wanted to know, whereas the neurologist kind of weaved around it a bit and left it open.

PD‐MCI_P5 (M, 64)

###### 3.1.2.4.3 Risk Reduction Strategies

While linking to support services was considered useful, advice on risk reduction strategies for dementia was perceived as important for participants with PD‐SCD.

I'm always for to do things and to find it out about risk of developing dementia, well what's going to happen to in the future, and so it's preparing you, if you could get or if you could be warned and there was something to do about it, something that could be done about it, then I would like to know.

PD‐SCD_P8 (F, 67)

###### 3.1.2.4.4 Advocating for the Person With PD

In line with carers' concern about the terminology used in the clinic to discuss cognitive impairment, participants agreed having someone present to advocate for the PwPD was crucial.

I think any person who receives a diagnosis like [dementia] has to have someone in their corner, you're constantly advocating for that person. When my mum was alive, she was the person. Could Dad have managed any of this process by himself? No way.

Carer‐PDD_P4 (PDD: M, 55; Carer: F, 55)

While carers emphasised the importance of advocating for PwPD and prioritising their comfort in clinical and personal decisions, they felt their discussions about cognitive decline of PwPD with clinicians should be approached carefully.

I find myself saying, “You know, dad, I just need to tell the doctor this. And I don't want you to be upset, but this is something I've noticed.” It's disrespectful. I wish that I could have an appointment with him [the clinician] by myself to go through it and say, “Hey, this is this”.

Carer‐PDD_P14 (Carer: F, 56; PDD: M, 71)

Carers highlighted the importance of clinicians seeking PwPD consent before discussing cognitive impairment, and dementia with the carer or support person in their presence.

Advocating for PwPD became even more critical as cognitive impairment advanced, complicating processes like advanced care planning and complex decision‐making. Support persons or family carers were seen as essential, given their deep understanding of PwPD, often positioning themselves as key decision‐makers in determining actions taken. The importance of involving a support person throughout the PD journey, particularly as cognitive decline progressed, was emphasised.

### 3.2 Stage 2: National Survey

#### 3.2.1 Sample Characteristics

Twenty‐five preliminary recommendations for neuropsychological evaluation, diagnosis, and post‐diagnostic care for cognitive disorders in PD were developed from the qualitative analysis of the focus group data (Stage 1). In total, 81 PwPD and carers participated in the national survey. The majority of respondents were PwPD (*n* = 69, 85%) and of these, close to two‐thirds (*n* = 44, 64%) had undergone a neuropsychological evaluation (PD‐SCD *n* = 28, PD‐MCI *n* = 8 12%, PDD *n* = 8, 12%). Table 3 summarises the demographic data of the participants.

**Table 3:** National survey participant characteristics (*N* = 81).

Characteristics		Numbers (%)	Duration since PD diagnosis^a^	
Participants	People living with Parkinson's disease (PwPD)	69 (85)	Range (years)	Numbers (%)
			0–4	18 (26)
			5–9	20 (29)
			10–14	16 (23)
			15–19	10 (12)
			20+	5 (6)
	Carers	12 (15)		
Gender	Female	45 (56)		
	Male	36 (44)		
Age range	< 50	4 (5)		
	50–59	11 (14)		
	60–69	26 (32)		
	70–79	27 (33)		
	80+	13 (16)		
State	Queensland	54 (67)		
	New South Wales	18 (22)		
	Victoria	5 (6)		
	Western Australia	2 (3)		
	South Australia	1 (1)		
	Australia Capital Territory	1 (1)		

a Duration of Parkinson's diagnosis as of 2025.

#### 3.2.2 Results

##### 3.2.2.1 Agreement Rating

All recommendations except one demonstrated agreement of 88% or more. The recommendation that a cognitive diagnosis should not be made on the same day as a neuropsychological assessment received the most varied ratings. It had a lower agreement rate (54%) while 21% of participants were unsure and 25% disagreed. The recommendations mapped to the identified themes and descriptive statistics of ratings are summarised in Table 4.

**Table 4:** National survey—Agreement ratings of recommendations by lived experience expert participants.

Recommendations	Source theme	Agree (%)	Disagree (%)	Unsure (%)	Median (IQR)
A brief cognitive screen should take place as soon as a diagnosis of Parkinson's disease is made.	Reluctance to assess	91	4	5	4 (1)
Any clinician, including allied health professionals (e.g., occupational therapist [OT], physiotherapist or speech pathologist), should be able to make a referral to a clinical neuropsychologist if there are concerns about the presence of cognitive changes.	Referral	94	1	5	4 (1)
Neuropsychological assessments should be undertaken on a recurring basis to monitor cognition.	Follow‐up assessment	85	4	11	4 (1)
Neuropsychological assessments should be undertaken in response to a noticeable change in cognition (e.g., planning, language, memory etc.) and/or noticeable change in other neuropsychological symptoms (e.g., depression, anxiety).	Referral	96	0	4	4 (1)
A diagnosis of mild cognitive impairment or dementia should not be given on the same day as a full neuropsychological assessment.	Delivery of assessment	54	25	21	4 (1)
When any diagnosis of mild cognitive impairment, or dementia is made, this should be delivered clearly, in a respectful manner.	More time to discuss and Focused discussion and monitoring	99	0	1	5 (1)
Clinicians should spend a considerable amount of time to clearly explain the diagnosis of a mild cognitive impairment or dementia.	More time to discuss	96	0	4	5 (1)
Clinicians should ask people with Parkinson's disease if they would like an in‐depth discussion about what a dementia diagnosis means for different areas of their life (such as the short, medium and long‐term outlook).	More time to discuss and Focused discussion and monitoring	94	1	5	5 (1)
People with Parkinson's disease who meet the criteria for a mild cognitive impairment diagnosis should be given a formal diagnosis, rather than ‘some cognitive decline’ or not being told about decline.	Transparency	91	0	9	4 (1)
Prior to a neuropsychological assessment, people with Parkinson's disease should be made aware of the purpose and potential outcomes of measures. Potential outcomes of neuropsychological evaluations include the possibility of a diagnosis of mild cognitive impairment or dementia, loss of legal or financial capacity, loss of capacity to drive.	Informed choice	99	1	0	5 (1)
Before a neuropsychological assessment, clinicians should discuss whether people with Parkinson's disease want to know the assessment outcome or whether this information should only be communicated with their carers or support people.	Consumer expert group (CCIG)	88	7	5	4 (1)
Before a neuropsychological assessment, clinicians should clarify contact details of other medical and allied health professionals of the person living with Parkinson's disease so that they can be informed of the outcomes of neuropsychological assessments.	Informed choice, role of support in diagnosis	95	1	4	4 (1)
Following a neuropsychological assessment, people with Parkinson's disease and their care partner/support person should be offered a feedback session to explain the outcomes of the assessment.	Advocating for PwPD	95	1	4	5 (1)
If a clinician asks a care partner or a support person about the current cognitive function of a person living with Parkinson's disease as part of the neuropsychological assessment, the person being assessed should be asked whether they want to be present during that conversation or not.	Advocating for PwPD	88	5	7	4 (1)
Following a neuropsychological assessment (regardless of the outcome), people should receive information to help reduce their future risk of dementia (e.g., general advice on exercise, diet, social and cognitive engagement, stress reduction, sleep etc.).	Risk reduction strategies	100	0	0	5 (1)
Following a diagnosis of mild cognitive impairment or dementia people should be given written materials or links to materials about these conditions, written in plain English.	Information in plain language	96	1	3	5 (1)
Following a diagnosis of dementia, people should be given contact details about where they can find more information or support (e.g., Dementia Australia).	Available support	97	1	3	5 (1)
After receiving a diagnosis of mild cognitive impairment or dementia, clinicians should be mindful to speak to the person with Parkinson's disease and their support person in consults and not exclude either person.	Advocating for PwPD/Role of support (person) in diagnosis	96	4	0	5 (1)
Telehealth‐based neuropsychological assessments should be made available to people who are unable to attend clinics in person (e.g., due to long travel times, mobility issues etc.)	Telehealth for cognitive assessment	92	4	4	5 (1)

## 4 Discussion

Current guidelines for cognitive disorders in PD lack input from people with lived experience. This study was therefore underpinned by a participatory approach that centred the voices and experiences of PwPD and their care partners. This paper aimed to develop consumer‐endorsed recommendations for evaluation, diagnosis, and post‐diagnostic care of cognitive disorders in PD. Twenty‐five recommendations were developed from exploratory qualitative analyses of focus group discussions, reflecting the perspectives on the healthcare experiences of individuals with cognitive disorders in PD and their carers. The recommendations were validated through a national survey of people with lived experience. Previous efforts to standardise care provision for people attending Australian memory clinics have resulted in best practice guidelines endorsed by lived experience experts [13]. In this study, we have developed recommendations for clinical services that PwPD attend, such as general neurology, geriatric and specialised movement disorders clinical services.

Perceived reluctance of healthcare professionals to formally assess cognition and unclear diagnostic pathways for PD‐MCI and PDD were shared frustrations among focus group participants. Lack of time, capacity, or knowledge may explain clinician reluctance to conduct cognitive evaluations for PwPD [22]. The differentiation between PD‐related cognitive impairment and other cognitive disorders is often poorly understood, even among healthcare professionals [22]. This is exacerbated by fluctuations in daily functioning, which can be both physical and cognitive, and the overlap of motor and non‐motor symptoms, further complicating the management of cognitive disorders in the clinic [23]. This complexity, along with stigma and lack of awareness in PwPD and caregivers, may lead to under‐recognition and under‐reporting of cognitive impairment and dementia in PD by clinicians [9, 23]. Improved recognition through increased awareness and knowledge of cognitive impairment and dementia among both clinicians and PwPD, as well as greater opportunities to identify these changes, is crucial for timely diagnosis [24].

The perceived lack of a clear pathway in terms of the progression of cognitive symptoms was consistent among participants. This led to uncertainty in the management of cognitive disorders, including issues such as underdiagnosis, difficulty planning for the future, and delayed access to relevant support services. Having a clear cognitive diagnosis was considered important by participants, consistent with broader dementia research [25]. A formal diagnosis is essential for ensuring optimal support and treatment [9]. Participants highlighted the value of having an advocate for the PwPD, particularly as communication and cognition began to decline, making it harder to express needs and navigate care. Previous research identified the onset of cognitive impairment, and dementia as a pivotal point for caregivers to transition into an advocacy role for PwPD [22]. Our findings align with this, reinforcing the importance of involving and supporting PwPD and their caregivers in decision‐making processes related to future planning and care management [26]. As PD progresses, the need for a support person becomes increasingly important, particularly in managing evolving challenges. Focus group participants also reported the lack of sufficient time in appointments, limited access to specialists, and feelings of being uninformed and unsupported, which aligns with findings from previous studies [9].

While the complexity of cognitive impairment in PD was acknowledged by focus group participants, discrete suggestions were made to improve the standard of clinical care. They emphasised the need for sufficient time dedicated to clear, transparent communication about cognitive symptoms and their impact on daily living during clinical consults. Sufficient time is needed for effective communication to identify individual needs, provide continuity of care by clinicians, and optimise the referral processes. However, as this is not possible in most clinical settings, PwPD should be referred to established community, government and not‐for‐profit services for people with cognitive disorders to gain a deeper understanding of their condition and receive adequate support. The findings of the present study also highlight the need to develop standardised protocols and guidelines to harmonise evaluation, diagnosis and management of cognitive disorders in PD, ensuring more consistent and comprehensive care. Standardising service provision and pathways of care can prevent delays in important planning discussions and access to healthcare services to ultimately improve the quality of life of PwPD and reduce the economic burden associated with PD‐MCI and PDD due to hospital admissions and long‐term care [7, 27, 28].

Our survey findings indicated strong consensus (> 85% agreement) among Australian PwPD and their carers on almost all recommendations derived from the focus groups. However, the recommendation on not to deliver a cognitive diagnosis on the same day as a full neuropsychological assessment was not endorsed (54% agreed, 21% uncertain). This may reflect concerns about the emotional and psychological impact of receiving a diagnosis without adequate time for processing or support [29, 30]. While timely diagnosis is valued, a same‐day approach may not always be feasible or preferred, emphasising the need for a personalised, tailored approach.

### 4.1 Clinical Implications

The inquiry of lived experience experts in this study aligns with the WHO recommendation to meaningfully engage individuals with lived experience in the development of policy, programs and services [12]. Our findings revealed gaps in the diagnostic and postdiagnostic pathways for cognitive disorders in PD from a consumer perspective and developed consumer‐derived recommendations endorsed by lived experience experts to bridge these gaps. These recommendations will contribute to best practice guidelines for the diagnosis, evaluation and management of cognitive disorders in PD, with the overall aim of improving the quality of care and quality of life for PwPD. They may help enhance clinical practice by supporting routine cognitive screening, standardising referral pathways, improving communication with PwPD and care partners, and encouraging multidisciplinary management, potentially reducing variability in care and improving patient outcomes.

### 4.2 Strengths and Limitations

A major strength of this study is the strong integration of lived‐experience perspectives, both through participant contributions and the CCIG that shaped aspects of the study process, ensuring the findings and recommendations are closely aligned with real‐world needs. While the impact of motor impairment is well recognised and reported for PD [23], the lived experience of cognitive impairment in PD is not often explored in the literature. This study adds to the limited literature exploring the experiences of PwPD in receiving evaluations, diagnoses, and care for their cognitive symptoms. Specifically, the Australia‐wide survey provides lived experience expert endorsement of recommendations for cognitive disorders in PD, an under‐explored yet important area of research. While online surveys may cause bias in favour of people who are active online, the study achieved a wide geographical representation of PwPD. Strong representation from PwPD is considered a strength of the study. However, carer representation was comparatively lower, likely due to the exceptional time constrains and unique barriers they face, more so than most other populations [31]. This study did not include PwPD without cognitive impairment, as the focus was on experiences related to cognitive assessment and management. Including this group in future research could provide insights into prevention and early support strategies.

Although a large proportion of survey respondents were from Queensland, participants were represented across multiple Australian states, providing some geographic diversity. We did not collect information on participation by First Nations peoples or other culturally and linguistically diverse groups, and therefore the applicability of the results to these populations cannot be determined. While the study offers valuable insights into the perspectives of PwPD and their care partners, caution is warranted when generalising the findings to other regions or cultural groups. Future research should aim to include participants from a broader cultural background to enhance generalisability.

A challenge specific to the focus groups was the communication difficulties faced by some PwPD (e.g., speech difficulty, language production and comprehension difficulty) who may not have been able to express their views as intended. Specialised communication training was provided to interviewers to mitigate this limitation and enable maximum participation by PwPD. Carer perspectives may have introduced potential bias in the focus groups. However, PwPD and carers were considered as a dyad, and their reports are presented collectively for the qualitative analysis to account for this bias. Although focus groups were planned to include four participants each, some were conducted with only two individuals across the PD cognitive subgroups (subjective decline, MCI, and dementia). While these smaller groups yielded rich individual insights, they may have reduced opportunities for group interaction and discussion.

While this study was conducted in Australia, the findings may have relevance for other countries with similar healthcare systems, particularly regarding the importance of standardised cognitive assessment, clear referral pathways, multidisciplinary care, and engagement with PwPD and care partners. Adaptation to local contexts would be required, but the principles outlined in the recommendations may inform the development of cognitive care practices internationally.

## 5 Conclusion

This study has developed consumer‐led recommendations to improve identification and management of cognitive disorders in PD, which were endorsed by lived experience experts across Australia. These results will inform the development of best practice guidelines to improve identification and management of cognitive disorders in PD, providing a lived experience perspective to improve standards of care for PwPD.

## Author Contributions

Study design and data collection were performed by Deepa Sriram, Daniel Bailey, Jihyun Yang, and Kirstine Shrubsole. Data analysis was completed by Deepa Sriram (Focus Group and Survey) and Daniel X Bailey (Focus Group). Preparation of the first draft was completed by Deepa Sriram, Deborah Brooks, Neil Page, Leander Mitchell, and Nancy. All authors contributed to writing – review and editing, conceptualization. Elton Lobo and Emily MCCann contributed to writing – review and editing. Nadeeka N. Dissanayaka led acquisition of funding for the project and contributed to writing – review and editing, conceptualization.

## Ethics Statement

This study was approved by the University of Queensland Human Research Ethics Committee (2023/HE002029) and Metro North Health HREC (HREC/2023/MNHA/100098).

## Consent

All participants of the focus groups and national survey provided informed consent.

## Conflicts of Interest

The authors declare no conflicts of interest.

## Data Availability

Data may be shared upon request.
